# Efficacy of an Imaging Anatomy Virtual Simulation Experiment Course in Undergraduate Medical Education: Nonrandomized Controlled Trial

**DOI:** 10.2196/80393

**Published:** 2026-05-13

**Authors:** ACeng Li, Lina Ma, Rui Peng, Juan Shi, Mingming Zhang, Minwen Zheng, Yayun Wang, Yayu Huang, Jing Ren

**Affiliations:** 1 Department of Radiology, Xijing Hospital, Air Force Medical University, No. 127, Changle West Road, Xi'an City, Shaanxi, 710032, China, 86 02984775424; 2 Teaching and Research Section of Human Anatomy and Histology and Embryology, Basic Medical College, Air Force Medical University, Xi'an City, Shaanxi, China; 3 National Basic Medical Experimental Teaching Demonstration Center, Basic Medical College, Air Force Medical University, Xi'an City, Shaanxi, China; 4 Internal Medicine Teaching and Research Office, Xijing Hospital, Air Force Medical University, Xi'an City, Shaanxi, China

**Keywords:** virtual simulation, human anatomy, undergraduate, undergraduate teaching, medical imaging

## Abstract

**Background:**

Human anatomy is the cornerstone of medicine. Currently, there is a lack of effective learning tools that can establish a correlation between 2D sectional anatomy and 3D stereoscopic anatomy and facilitate the conversion between the two.

**Objective:**

This study aimed to evaluate the efficacy of the Imaging Anatomy Virtual Simulation Experiment (IAVSE) course in undergraduate education.

**Methods:**

This nonrandomized controlled trial was conducted with 102 third-year medical students from the 2017 and 2018 cohorts: 50 medical students who elected to take the IAVSE course were assigned to the experimental group, and 52 students who took the traditional laboratory course served as the control group. Primary outcomes assessed by teaching evaluation included the third-year human anatomy theory scores and fourth-year medical imaging theory, practical test, and total scores. The secondary outcome was a 7-item Likert scale questionnaire based on Bloom’s Taxonomy of Educational Objectives. Statistical analyses were performed using SPSS (IBM Corp), with the independent samples *t* test applied for normally distributed continuous data and the Mann-Whitney *U* test for nonnormally distributed data. A two-tailed *P*<.05 was considered statistically significant for all tests.

**Results:**

The results of the teaching evaluation indicated a statistically significant difference between the experimental group and the control group. The experimental group achieved higher scores in human anatomy theory (experimental group: median 85, IQR 84.25‐85.50, control group: median 81.75, IQR 78.13‐83.00; *P*<.001), medical imaging theory (experimental group: median 61.03, IQR 60.41‐61.53, control group: median 59.03, IQR 55.66‐59.53; *P*<.001), practical testing (experimental group: median 22.50, IQR 21.50‐23.00, control group: median 20.50, IQR 19.00‐22.00; *P*<.001), and total scores (experimental group: mean 83.26, SD 2.58; control group: mean 78.46, SD 3.76; *P*<.001). Student feedback collected via a Likert questionnaire also revealed significantly higher ratings in the experimental group across multiple domains, including enjoyment, interactivity, participation, satisfaction, learning efficiency, usability, and acceptance (all *P* value*s* <.001, except for serviceability, *P*=.02). Furthermore, the experimental group demonstrated a higher level of acceptance toward the virtual simulation course.

**Conclusions:**

The IAVSE course effectively bridges the gap between human anatomy and medical imaging. It enhances students’ spatial understanding and academic performance and stimulates their learning interest. It thus holds significant potential for broader application in undergraduate medical education.

## Introduction

The study of human anatomy is the cornerstone of medical education [1]. Through theoretical instruction, students develop a foundational understanding of the composition and morphological structure of human organs. Practical courses, particularly dissection courses, play a crucial role in anatomical teaching by facilitating the integration of theoretical knowledge with hands-on experiences [2,3].

However, traditional approaches to teaching human anatomy have primarily focused on general 3D anatomical structures, often overlooking the importance of 2D cross-sectional anatomy [4]. To understand the spatial relationships between different organs, students are required to develop a strong 3D spatial imagination [5,6]. This presented a significant challenge, especially when dealing with complex anatomical structures and their interrelationships [7]. As students progress to medical imaging in subsequent years, many find it difficult to translate 2D images into a coherent 3D understanding [8]. This difficulty is particularly pronounced among students with limited spatial visualization skills, adversely impacting their overall learning outcomes [9].

Recently, emerging virtual simulation technologies comprising augmented reality, mixed reality, and virtual reality (VR) have gained prominence in higher education driven by increased accessibility and affordability [10,11]. This technological advancement has prompted educational institutions to initiate research and development projects assessing their pedagogical efficacy when integrated into existing curricula [12]. A substantial body of evidence indicates that integrating these innovative approaches with conventional teaching methods significantly enhances the effectiveness of anatomy education for both instructors and learners [13]. As demonstrated in relevant studies, 3D visualization serves as a valuable educational tool, particularly in improving learning outcomes, by facilitating the mental representation of anatomical structures, a cognitive process essential for consolidating long-term knowledge [14,15]. Specifically, from a technical perspective, virtual simulation refers to the spatial superimposition of interactive virtual objects onto physical environments. Within medical education, this technology is being progressively adopted to provide multidimensional, intuitive learning resources that transform abstract concepts into tangible 3D models, thereby creating immersive learning experiences. These experiences are hypothesized to promote deeper understanding and knowledge retention [16]. Consequently, student enthusiasm for 3D visualization technologies in anatomical studies has increased notably, a trend mirrored by the booming market for anatomy-specific 3D learning tools [17].

This study was designed to address the critical gap between human anatomical knowledge and medical imaging interpretation. To this end, we developed the Imaging Anatomy Virtual Simulation Experiment (IAVSE) course, which specializes in imaging anatomy. This research is particularly significant as it systematically compares virtual simulation–based pedagogy with conventional didactic approaches within undergraduate medical education, using a dual evaluation framework that integrates objective competency assessments with comprehensive subjective feedback. Based on the core objectives of this study, the following research hypotheses are proposed for empirical investigation:

The VR-based IAVSE course functions as an effective transitional teaching tool connecting human anatomy and medical imaging in undergraduate medical education, resulting in improved performance in human anatomy and medical imaging assessments, heightened spatial reasoning ability, and greater knowledge retention.

## Methods

### IAVSE Course

#### Development Process of IAVSE Course

**Figure 1.**
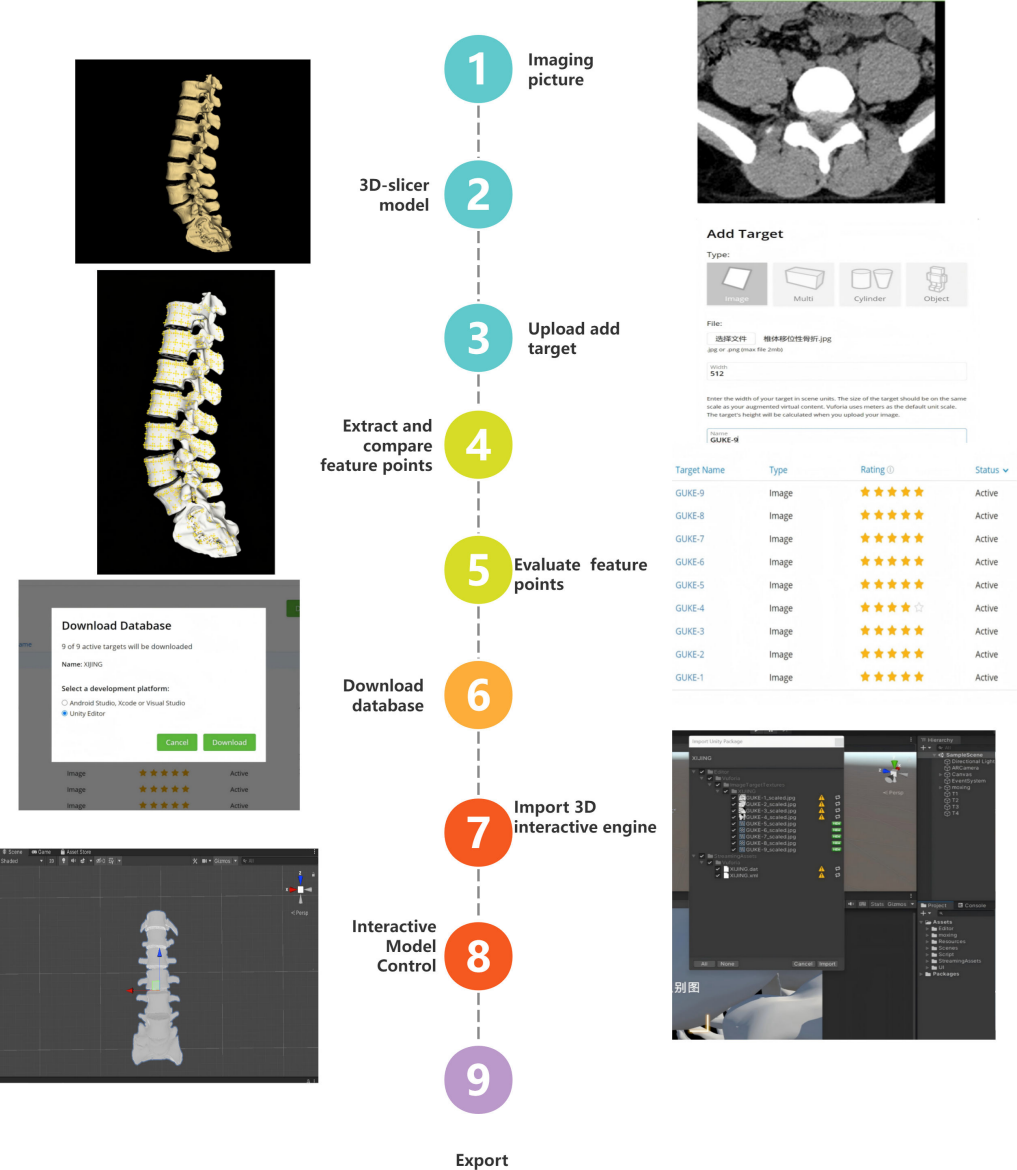
Development process of the Imaging Anatomy Virtual Simulation Experiment course.

The IAVSE course was collaboratively developed by the Human Anatomy Teaching and Research Section following a methodologically rigorous workflow (Figure 1). To meet the project requirements, Vuforia (Engine 9.x; PTC Inc), an industry-leading augmented reality engine, and Unity (2019.4 LTS), a robust application development platform, were selected as the foundational technologies. C# was used as the primary programming language for scripting interactive logic.

##### Data Curation Phase

First, for case selection, clinically representative computed tomography or magnetic resonance imaging thin-section (<1 mm) imaging data were acquired from standardized diagnostic repositories.

Second, for preprocessing, all clinical computed tomography or magnetic resonance imaging datasets used in this study underwent a thorough deidentification process to strictly protect patient privacy. Specifically, before these datasets were used for course development, the professional software (RadiAnt DICOM Viewer; Medixant Sp. z o.o) had completely removed or anonymized all personal identifiable information of patients, including names, medical record numbers, dates of birth, and any other information that could be used to identify patients. Digital Imaging and Communications in Medicine datasets were exported with optimized windowing parameters.

##### 3D Reconstruction Pipeline

First, for volumetric modeling, raw imaging data were processed using 3D Slicer v4.11 to generate surface-rendered anatomical models.

Second, for mesh optimization, models underwent topology-preserving decimation (polygon reduction >70%) and Laplacian smoothing for computational efficiency.

##### Model Target Preparation With Vuforia Engine 9.x

First, the optimized 3D model was uploaded to the Vuforia Developer Portal to create a model target, which served as a unique digital fingerprint for subsequent spatial registration in the VR teaching system.

Second, for algorithm implementation, a natural feature recognition algorithm (eg, scale-invariant feature transform variant) was used to extract scale-invariant key points (minimum 200 points/image). In tandem, the model target generator tool automatically extracted a dense set of scale-invariant geometric features (including vertex positions and normals) from the model’s surface mesh, and these multidimensional features together laid a solid foundation for precise virtual model placement upon real-time recognition. Based on the extracted key points and geometric features, feature point libraries were constructed through nearest-neighbor ratio testing (threshold: 0.7); the random sample consensus algorithm was further applied for outlier rejection to eliminate false matching points caused by environmental interference, ensuring the accuracy of feature matching for spatial registration.

Third, for feature point stability and model target quality evaluation, the quality and robustness of the generated model target were rigorously evaluated to substantiate technical performance, with the model target generator tool ensuring uniform feature coverage across the model surface. Robustness was additionally tested against simulated variations in lighting and partial occlusion (typical real-world application interferences). For formal deployment, all model targets were required to meet strict quantitative metrics (core validation for engineering contributions), as follows:

- Vuforia star rating: ≥3 stars (a composite metric assessing feature quality, spatial distribution, and model complexity, ranging from 1 to 5 stars)
- Key point matching rate: >80% during runtime testing
- Tracking loss rate: <5 instances of continuous tracking loss per minute under standard experimental conditions.

Fourth, the finalized model target database, containing the model’s feature descriptors, was downloaded for integration into the Unity development environment.

##### VR Application Development in Unity

The interactive VR learning module was developed in Unity 3D Engine (v2019.4). The downloaded Vuforia target database was imported, and the anatomical model was configured as a trackable target. Initial prototype testing confirmed a model tracking confidence level of ≥80%.

First, for XR platform configuration, the application was built for the Meta Quest 2. The Oculus XR Plugin (Unity Technologies), Vuforia (Engine 9.x), and Oculus Integration SDK (Meta Platforms) were installed via Unity’s Package Manager. Player settings were configured for the Android platform with a minimum application programming interface level of 24 (Android 7.0 “Nougat”) to match the Meta Quest 2 system requirements.

Second, for scene implementation, a VR camera rig was configured for stereoscopic rendering. The anatomical model was linked to the Vuforia Model Target behavior to ensure stable spatial registration. Single-pass stereo rendering was enabled to optimize graphical performance and frame rate stability.

##### Interaction Programming (C#)

First, for model manipulation, the users could grab, rotate, and translate the model using Oculus Touch controllers.

Second, for progressive disclosure, anatomical structures were organized in layers, allowing users to toggle their visibility incrementally via the controller trigger.

Third, for interactive labeling, mesh colliders were added to key structures. Selection was enabled via a controller ray cast, triggering the display of dynamic 3D text annotations at the hit point.

Fourth, for control scheme, the controller joystick was mapped to model rotation and translation, the trigger button cycled through anatomical layers, and the grip/side button reset the model to its initial position.

### Infrastructure Configuration

**Figure 2.**
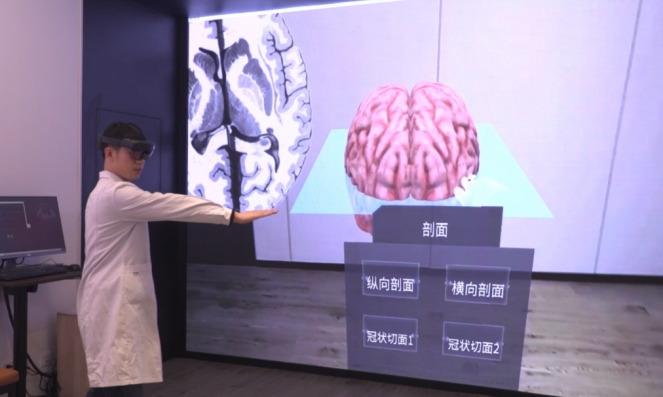
The classroom scene of the virtual simulation experiment in image anatomy focusing on the interaction between 3D anatomy and 2D tomographic images, incorporating game-based assessments for upgrades.

The hardware platform consists of Meta Quest 2 stand-alone head-mounted displays with inside-out 6-degree-of-freedom tracking (Figure 2).

#### Interaction Paradigm

The interactive operation of VR devices can be reflected in three aspects: planar manipulation, volumetric exploration, and multimodal feedback. Specific details are provided below.

1. Planar manipulation: hand gestures (via optional bare-hand tracking or touch controllers) enable interaction with virtual menus and 2D interfaces.
2. Volumetric exploration: 6-degree-of-freedom manipulation enables real-time translational, rotational (360° axis-free), and translational adjustments.
3. Multimodal feedback: built-in 3D spatial audio and haptic feedback (via touch controllers) provide immersive sensory experiences, with haptic vibrations offering tactile confirmation for in-game interactions.

### Study Design

#### Participant Recruitment and Group Allocation

A nonrandomized prospective cohort study was conducted, enrolling 102 third-year students from the 2017 and 2018 cohorts of the 8-year clinical medicine program at Air Force Medical University. During the human anatomy course, the IAVSE program was offered as an elective curriculum innovation.

Participants were allocated into two groups based on their course selection: (1) experimental group (n=50), with 20 students from the 2017 cohort and 30 from the 2018 cohort who elected to take the IAVSE course, and (2) control group (n=52), with 32 students from the 2017 cohort and 20 from the 2018 cohort who received the traditional laboratory course.

To enhance comparability between the groups, 2 key methodological safeguards were implemented:

1. Temporal alignment: both groups proceeded to complete an identical, standardized medical imaging curriculum in their subsequent (fourth) academic year.
2. Instructor consistency: the same teaching faculty supervised and delivered the imaging curriculum for both cohorts.

The sample size of this study was determined by the actual number of students who voluntarily selected the new-type elective course, which was uncontrollable in practice. Specifically, the experimental group consisted of 50 participants and the control group consisted of 52 participants, with a total sample size of 102. Due to the voluntary nature of the elective course, it was not feasible to predetermine or expand the sample size based on a priori power analysis, as student participation depended entirely on the actual registration situation of the new elective course. To verify the statistical power of the study, a post hoc power analysis was performed using G*Power 3.1 (Heinrich Heine University Düsseldorf) software. The results showed that with a total sample size of 102, a medium effect size of 0.5 (commonly used in educational intervention studies), and a significance level (α) of .05, the statistical power (1-β) of the study reached .81, which is higher than the commonly accepted threshold of .8. This indicates that the study had sufficient statistical power to detect statistically significant differences in learning outcomes between the 2 groups.

#### Third-Year Curriculum Framework

The third-year human anatomy curriculum for both cohorts was based on the textbook *Human Anatomy (Third Edition)* [18]. The curriculum was structured around two components:

1. Traditional theoretical instruction: a 60-hour theoretical course covering human anatomy.
2. Laboratory courses: totaling 40 hours. The laboratory component differed between groups. The control group completed a 40-hour traditional cadaver-based laboratory course. In contrast, the experimental group undertook a combined laboratory module, consisting of 24 hours of traditional cadaver-based sessions plus 16 hours of IAVSE course. All laboratory sessions were delivered synchronously with the theoretical lectures.

It is particularly noteworthy that due to the COVID-19 pandemic beginning in 2019, the school adjusted the teaching arrangements for key courses. The courses covered in this study for the 2017 and 2018 cohorts were delayed by 6 months. The 2017 cohort’s human anatomy course commenced instruction in November 2020.

#### Control Group Pedagogy (Year 3)

The control group received the following standardized instructions are provided through: the following:

1. Traditional theoretical instruction includes syllabus-compliant PowerPoint presentations containing identical theoretical content and contact hours as the experimental group.

2. The laboratory course (traditional cadaver-based laboratory course, 40 hours) includes the following:

- Guided Ddemonstrations: Ffaculty-led reviews of key anatomical relationships.
- Active Llearning: Rrotation through prosected specimens and dissection stations and access to a video atlas system.
- Collaborative Llearning: Sstructured small-group discussions and instructor-facilitated question and answer Q&A sessions.
- Guided demonstrations: faculty-led reviews of key anatomical relationships
- Active learning: rotation through prosected specimens and dissection stations and access to a video atlas system
- Collaborative learning: structured small-group discussions and instructor-facilitated question and answer sessions

3. Independent Sstudy: includes Ppost-class review using textbook and digital slides, complemented by weekly self-assessment quizzes.

#### Experimental Group Intervention (Year 3)

The curriculum framework of the experimental group was as follows:

1. Traditional theoretical instructions are identical to the control group.
2. The laboratory course included 24 hours of traditional cadaver-based laboratory course plus 16 hours of IAVSE course. The IAVSE was designed as follows:
  - XR infrastructure conducted using Meta Quest 2 VR headsets, encompassing 6 one-credit modules covering major anatomical systems.
    Gamified learning program includes the following:
  -  Core mechanics: real-time 3D model manipulation (rotation, zoom, and slicing) and anatomical “jigsaw puzzles” with haptic feedback
  -  Formative assessment: competency checks using a composite scoring algorithm, including spatial reasoning tasks with automated feedback.

Due to the limited availability of only 10 Meta Quest 2 units, it was not feasible for all participants in the experimental group to undergo VR training simultaneously. A total of 20 students from the 2017 cohort were divided into 2 groups, and 30 students from the 2018 cohort into 3 groups. To address this equipment constraint while preserving the statistical power of the sample size, a staggered group training protocol was adopted. Each VR training session was scheduled within 1 day of the corresponding theoretical course to maintain learning continuity. The entire VR-based IAVSE course was delivered over 7 to 8 weeks, with 2 to 3 training hours per week. All procedures, including preparatory guidance, hardware setup, and software navigation, were strictly standardized and administered by the same instructors to ensure instructional consistency and experimental reliability across all subgroups

1. Independent learning is also identical to the control group.

#### Fourth-Year Curriculum (Both Groups)

In the subsequent academic year, both cohorts transitioned to the same standardized medical imaging curriculum.

The core text used was the eighth edition of *Medical Imaging* [19].

Instructional standardization included (1) faculty with ≥5 years’ specialized teaching experience and (2) system-based curriculum covering.

#### Teaching Evaluations Protocol

##### Human Anatomy Evaluation (Year 3)

Theoretical examination was administered postcurriculum completion, aligned with syllabus objectives. The following are the key parameters:

1. Total score: 100 points, difficulty index: 0.72 (calculated via pretest calibration)
2. Question types: multiple-choice (60 items, 1 point each) and short answer (4 structured questions, 10 points each)
3. Time allocation: 60 minutes
4. Content focus: core anatomical relationships and systemic integration

##### Medical Imaging Evaluation (Year 4)

A 2-stage competency assessment was implemented:

1. Theoretical examination (70 points):
  - Format: 35 multiple-choice questions (2 points each)
  - Duration: 25 minutes
  - Scope: radiographic principles and cross-sectional anatomy

1. Practice testing (30 points):
  - Task: diagnostic report writing for 3 clinical cases (10 points each)
  - Duration: 15 minutes per task
  - Evaluation criteria: diagnostic accuracy (40%), anatomical correlation (30%), and clinical justification (30%)

##### Student Feedback

To complement the objective assessments, subjective student perceptions were coll

ected using a purpose-built questionnaire. All participants were asked to complete a 5-point Likert scale questionnaire (1=Strongly disagree, 5=Strongly agree) based on Bloom’s Taxonomy of Educational Objectives [20,21]. The 7-item questionnaire includes (1) intrinsic motivation (enjoyment and satisfaction), (2) interactive engagement (participation, interactivity, and acceptance), and (3) pedagogical efficacy (learning efficiency and usability).

The questionnaire comprised 7 items designed to measure 3 key dimensions derived from the cognitive, affective, and psychomotor domains of Bloom’s taxonomy:

- Cognitive domain: items assessed learning efficiency (related to comprehension, application, and analysis) and the usability of the tool (related to synthesis and evaluation).
- Affective domain: items assessed enjoyment (reflecting the receiving and responding levels) and overall satisfaction with the course (reflecting the valuing level).
- Psychomotor domain: items assessed participation (perception and set) and the quality of interactivity (guided and complex response). An item on course acceptance served as a composite measure of students’ recognition of the interactive format and content.

The questionnaire was administered to students in both the experimental and control groups immediately following the intervention period.

### Statistical Analysis

Statistical analysis was carried out by SPSS 26.0 (IBM Corp). The data distributions were assessed with the Kolmogorov-Smirnov test. The measurement data following normal distribution were expressed as mean (SDs), and the comparison between the groups was performed using 2 independent sample two-tailed *t* tests. Conversely, the measurement data that deviated from normal distribution were expressed as medians (IQRs), and Mann-Whitney *U* tests were used for group comparisons. Count data were presented as frequency and percentage (n/N, %), and chi-squared tests were applied for group comparisons. For the student feedback questionnaire, the internal consistency of the Likert scale items was assessed using Cronbach α, with a coefficient >.70 considered acceptable. The suitability of the data for exploratory factor analysis was evaluated using the Kaiser-Meyer-Olkin measure, with a value >0.60 deemed acceptable. A 2-tailed *P*<.05 was considered statistically significant for all tests.

### Ethical Considerations

This study enrolled undergraduate medical students as participants. According to the Measures for the Review of Science and Technology Ethics (for trial implementation) (Guoke Fa Jian [2023] No. 167) issued by the Ministry of Science and Technology of the People’s Republic of China [22], scientific and technological activities posing no more than minimal risk—defined as routine risks in daily life or risks equivalent to standard health checkups—qualify for simplified reviews or ethical exemption. The Measures for the Ethical Review of Life Science and Medical Research Involving Humans, issued by the Department of Science, Technology and Education of the National Health Commission [23], further clarifies that research using anonymized data that cause no bodily harm and exclude sensitive personal information is eligible for ethical review exemption. As a routine educational and teaching practice devoid of invasive interventions, this study fully meets the aforementioned exemption criteria; moreover, it aligns with the Charter of the Medical Ethics Committee of Air Force Medical University [24], which prioritizes the protection of participants’ dignity, safety, and rights, and explicitly exempts routine medical teaching research, standard teaching practices, curricular interventions, and teaching efficacy evaluations from the full ethical review.

This research was conducted as an integral component of routine undergraduate medical education, with all data anonymized and deidentified prior to analysis and no personally identifiable information (eg, full names and student identification numbers) collected in strict adherence to the privacy and data protection provisions outlined in the aforementioned national regulations and institutional charter. Informed consent was waived given the study’s integration into routine teaching activities, and complete data deidentification guaranteed the full protection of participants’ legitimate rights and interests, consistent with the core ethical principles of respect for autonomy and risk minimization.

## Results

### Baseline Demographic Characteristics

The experimental (n=50) and control (n=52) groups demonstrated demographic equivalence, with no statistically significant intergroup differences in age or gender distribution. There was no statistically significant difference between the first- and second-year grade point averages of the 2 groups (*P*>.05). This demographic parity confirmed the groups’ comparability for subsequent pedagogical comparisons (Table 1).

**Table 1.** Comparison of basic information of the 2 groups.

Project	Experimental group (n=50)	Control group (n=52)	*P* value
Sex ratio (female:male)	23:27	28:24	.42^a^
Age (y), median (IQR)	21 (20‐22)	21 (20‐23)	.45^b^
GPA1^d^, mean (SD)	3.45 (0.23)	3.47 (0.21)	.07^c^
GPA2^e^, mean (SD)	3.51 (0.20)	3.51 (0.17)	.44^c^

a Chi-square test.

b Wilcoxon Mann-Whitney *U* test.

c *t* test.

d GPA1: first-year grade point average.

e GPA2: second-year grade point average.

### Teaching Evaluation

#### Human Anatomy Theoretical Assessment

**Figure 3.**
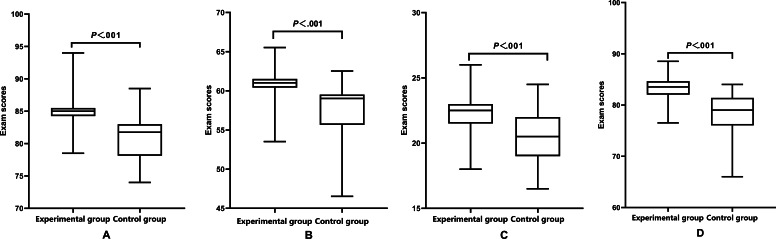
Comparison of (A) human anatomy theory assessment, (B) medical imaging theory assessment, (C) practical testing, and (D) total scores of the 2 groups.

The theoretical assessment scores of the experimental group were higher than those of the control group (median 85.00, IQR 84.25‐85.50 vs median 81.75, IQR 78.13‐83.00), and the difference was statistically significant (*P*<.001, Figure 3).

#### Medical Imaging Competency Evaluation

Following the completion of fourth-year medical imaging coursework, the experimental group achieved higher scores in theoretical assessment, practical testing, and total scores compared to the control group, with all differences reaching statistical significance (*P*<.001, Table 2 and Figure 3).

These significant statistical differences align with the study’s core hypothesis, indicating that the IAVSE course effectively boosts core spatial reasoning ability for anatomical learning and offers direct quantitative evidence for its application in anatomical education.

**Table 2.** Comparison of medical imaging theory assessment, practical testing, and total scores of the 2 groups (points).

Project	Experimental group (n=50)	Control group (n=52)	*P* value
Theoretical examination, median (IQR)	61.03 (60.4‐61.53)	59.03 (55.66‐59.53)	<.001^a^
Practice testing, median (IQR)	22.50 (21.50‐23)	20.50 (19‐22)	<.001^a^
Total points, mean (SD)	83.26 (2.58)	78.46 (3.76)	<.001^b^

a Wilcoxon Mann-Whitney *U* test.

b *t* test.

#### Student Feedback

The survey achieved 100% response validity (102/102). As assessed by the Kolmogorov-Smirnov test, responses within each group deviated from a normal distribution (*P*<.05); therefore, response data are summarized using medians (IQRs).

Following the analytic procedures outlined in the *Methods* section, the psychometric properties of the questionnaire were evaluated. The instrument demonstrated high reliability, with an overall Cronbach α=0.883. Validity assessment for factor analysis confirmed data suitability, yielding a Kaiser-Meyer-Olkin measure of 0.820 and a significant Bartlett test of sphericity (*P*<.001).

**Figure 4.**
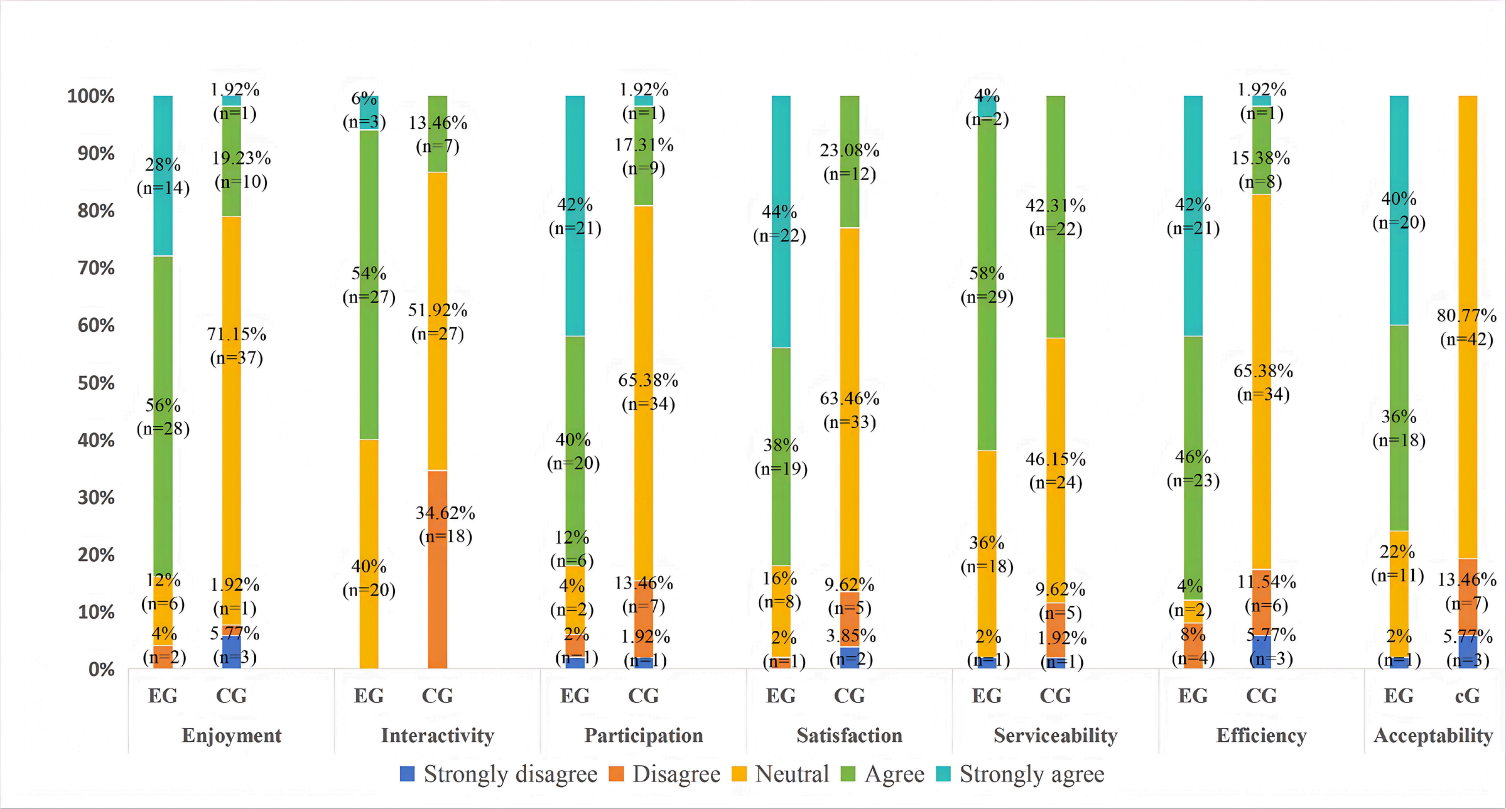
Evaluation of human anatomy. CG: control group; EG: experimental group.

The comparative analysis of 5-point Likert scale responses revealed statistically significant advantages for the experimental group across 7 pedagogical dimensions (Table 3 and Figure 4). Such positive subjective feedback covering the cognitive, affective, and psychomotor domains indicates that the course optimizes learning experiences and facilitates competency development, thereby laying a solid foundation for better adapting to clinical practice requirements and enhancing clinical reasoning abilities.

**Table 3.** Evaluation of human anatomy (points).

Project	Experimental group (n=50), median (IQR)	Control group (n=52), median (IQR)	*Z* value	*P* value
Interesting	5 (4‐5)	3 (3‐3)	6.11	<.001^a^
Interactivity	4 (3‐4)	3 (2‐3)	5.74	<.001^a^
Degree of participation	4 (4‐5)	3 (3‐3)	6.13	<.001^a^
Degree of satisfaction	4 (4‐5)	3 (3‐3)	6.43	<.001^a^
Serviceability	4 (3‐4)	3 (3‐4)	2.37	.02^a^
Learning efficiency	4 (4‐5)	3 (3‐3)	6.56	<.001^a^
Acceptance level	4 (3.75‐5)	3 (3‐3)	7.41	<.001^a^

a Wilcoxon Mann-Whitney *U* test.

## Discussion

### IAVSE Course as an Effective Bridge Program

This study pioneered the integration of a novel IAVSE course into the undergraduate medical curriculum. Using a mixed methods approach that combined objective instructor assessments with subjective student feedback, we evaluated the course’s efficacy from complementary perspectives. Our findings indicate that implementing the IAVSE course following human anatomy instruction (1) was associated with significantly enhanced student performance in human anatomy and improved subsequent academic achievement in the fourth-year medical imaging course and (2) appeared to effectively foster students’ comprehension of complex spatial relationships and their ability to mentally reconstruct 3D anatomy from 2D sectional images. Additionally, the course was linked to increased student engagement and self-reported learning efficacy. Taken together, these results suggest that the IAVSE course can serve as a valuable pedagogical bridge between human anatomy and medical imaging, highlighting its potential to enhance undergraduate medical education.

### Synergistic Enhancement of Anatomical Understanding

The experimental group demonstrated statistically superior performance in human anatomy assessments (*P*<.01), validating the efficacy of the IAVSE course. This improvement can be attributed to 3 key mechanisms as follows:

1. Spatial cognition reinforcement: the IAVSE enables multiplanar manipulation of 3D anatomical models, facilitating mental rotation skills essential for understanding structural relationships.
2. Procedural memory formation: real-time virtual dissection exercises promote kinesthetic learning, aligning with evidence that haptic-enhanced simulations improve long-term knowledge retention by 23% to 28% compared to passive observation [25,26].
3. Collaborative learning optimization: the shared virtual environment fosters peer-to-peer knowledge exchange, addressing the specimen access limitations noted in traditional curricula [27,28].

Notably, our results extend previous findings by demonstrating a 15% to 20% performance gap in neurovascular anatomy modules—a critical predictor of surgical aptitude [29,30]. This advancement stems from the course’s unique capacity to visualize deep anatomical strata through virtual layering techniques, overcoming the planar limitations of cadaveric dissection [31].

### Foundational Preparation for Medical Imaging Competency

The results of this study show that the elective IAVSE course markedly improves the teaching efficacy of medical imaging in subsequent courses. This is demonstrated by the higher theory, practice, and total scores in medical imaging among fourth-grade students in the experimental group compared to those in the control group. Being proficient in understanding 2D tomographic anatomy, which entails grasping the organ and tissue structure, location, and adjacent morphology in sagittal and coronal sections, is essential for mastering medical imaging. However, traditional human anatomy courses mainly focus on 3D gross anatomical structures. As a result, when students first encounter medical imaging, they face difficulties in transforming these abstract 2D tomography images into a comprehensive understanding due to this disparity [32]. This challenge has persisted for many years and contributes to students’ limited comprehension of medical imaging concepts. To tackle the teaching challenges, the team carried out a virtual simulation experiment in the image anatomy class. This approach was designed to enhance students’ understanding of 3D anatomy and their interaction with 2D tomograms through virtual means [33]. Additionally, a game-based upgrade assessment was introduced to facilitate progress to subsequent questions and formative assessments. By synchronously mastering tomography anatomy during human anatomy learning, students were able to establish a solid foundation for future medical imaging courses, leading to significant enhancements in academic outcomes.

### Demographic-Tailored Engagement Strategy

In comparison with the control group, the questionnaire results revealed a substantial improvement in students’ interest, interactivity, participation, satisfaction, learning efficiency, availability, and acceptance in the experimental group. These findings suggest that the implementation of a virtual simulation experiment course on image anatomy effectively contributes to enhancing students’ learning efficiency and interest [34]. Since 2018, the post-2000 generation has become the dominant demographic on university campuses. Having grown up in the information technology era, they possess active cognitive abilities and show a greater inclination to embrace novelty. The integration of simulation-based teaching methods has significantly enhanced students’ learning interests by providing immersive experiences [35]. Incorporating game elements into courses, where progression to subsequent levels depends on correctly answering previous questions, increases student engagement and fosters a profound sense of accomplishment during knowledge acquisition. As a result, this approach makes the learning process more relaxed and enjoyable while stimulating overall positivity, increased satisfaction with learning outcomes, self-efficacy beliefs, and engagement with immersive technologies. It is noteworthy that the enrollment for the virtual simulation experiment course on image anatomy in 2018 saw a significant increase compared to that in 2017 (30 vs 20). This surge can be largely attributed to positive feedback and recommendations from previous students. Furthermore, it is worth mentioning that students from the 2019 and 2020 cohorts accounted for over 80% of the total enrollment, further validating their strong affinity for this course [36].

### Limitations

While demonstrating pedagogical efficacy, this study presents key methodological constraints:

1. Generalizability limitations: the single-center cohort (N=102) restricts statistical power for subgroup analyses and generalizability. Institution-specific factors and cohort characteristics (eg, baseline digital literacy and learning preferences) may have constrained the applicability of results to other educational settings.
2. Design complexity: the conflation of VR simulation with traditional instruction prevented the isolation of the unique contribution of the VR component to the observed outcomes, leaving its specific mechanistic role unclear.
3. Temporal constraints: the 2-year evaluation window insufficiently captures longitudinal skill retention beyond undergraduate training.
4. Selection bias risk: as this was a newly developed elective course, and student choice was fully respected in enrollment, a nonrandomized study design was necessarily adopted, which may have introduced selection bias. Although baseline comparisons of gender, age, and prestudy GPA indicated acceptable group homogeneity, potential differences in learning motivation, digital literacy, and interest in anatomy were not evaluated using standardized questionnaires, and such pre-existing differences could have influenced the study outcomes.
5. Implementation constraints: the development of VR-based courses involves notable technical complexity and initial financial investment. Although VR may offer long-term cost and adaptability advantages [37], these initial barriers remained significant for widespread adoption during the study period.
6. A priori power analysis: it should be noted that no a priori power analysis was conducted in this study, which is mainly due to the uncontrollable sample size―this study focused on a new-type elective course, and the sample size was determined by the number of students who voluntarily registered, making it impossible to pre-estimate the sample size based on the power analysis. However, we supplemented a post hoc power analysis in the revised manuscript, and the results confirmed that the study had sufficient statistical power (1-β=.81) to detect significant differences in learning outcomes, which effectively mitigates this limitation.

### Future Directions

The large-scale, multicenter randomized controlled trials (recruiting ≥500 participants across diverse institutional settings) should be conducted to strengthen the evidence base and enhance generalizability. Future studies with a randomized controlled design and comprehensive baseline psychological assessments are warranted to further validate the efficacy of the VR-based IAVSE program. Independent virtual teaching stations for comparison with traditional lecture stations should be established to isolate the impact of core teaching elements and identify the effective components of interventions. Longitudinal follow-up should be extended to students’ entry into clinical residency training and their long-term professional development to assess the sustainability of educational outcomes. Subsequently, a comprehensive VR learning platform will be developed to integrate anatomical knowledge with clinical multidisciplinary modules and establish a complete learning framework, thereby more directly fostering students’ core competencies, such as spatial reasoning ability and diagnostic acuity [38].

### Conclusions

The IAVSE course represents a novel endeavor, serving as a vital conduit between human anatomy and medical imaging. It effectively addresses the interdisciplinary integration of these 2 domains and the inherent challenges therein. The primary objectives of this initiative are twofold: to enhance academic performance in human anatomy and to facilitate students’ transition from visualizing 3D anatomical images to comprehending abstract 2D cross-sectional images by strengthening their spatial cognitive abilities. Consequently, this approach significantly improves the instructional efficacy of human anatomy and medical imaging courses while concurrently fostering students’ learning interest. In turn, it creates a more engaging and enjoyable educational environment, conducive to in-depth knowledge acquisition. Ultimately, this innovative approach provides fresh insights and perspectives, offering valuable implications for advancing medical education through the integration of immersive simulation technologies.

## Abbreviations

IAVSE: Imaging Anatomy Virtual Simulation Experiment
VR: virtual reality
